# Multidisciplinary investigations of the diets of two post-medieval populations from London using stable isotopes and microdebris analysis

**DOI:** 10.1007/s12520-019-00910-8

**Published:** 2019-08-16

**Authors:** Madeleine Bleasdale, Paola Ponce, Anita Radini, Andrew S. Wilson, Sean Doherty, Patrick Daley, Chloe Brown, Luke Spindler, Lucy Sibun, Camilla Speller, Michelle M. Alexander

**Affiliations:** 1 Department of Archaeology, Max Planck Institute for the Science of Human History, Jena, Germany; 2 BioArch, Department of Archaeology, University of York, York, UK; 3 PalaeoHub, Department of Archaeology, University of York, York, UK; 4 School of Archaeological & Forensic Sciences, University of Bradford, Bradford, UK; 5 Department of Archaeology, University of Exeter, Exeter, UK; 6 Oxford Radiocarbon Accelerator Unit, University of Oxford, Oxford, UK; 7 Archaeology South-East, Institute of Archaeology, University College London, London, UK; 8 Department of Anthropology, University of British Columbia, Vancouver, Canada

**Keywords:** Diet, Collagen, Isotopes, Hair, Calculus, Post-medieval

## Abstract

This paper presents the first multi-tissue study of diet in post-medieval London using both the stable light isotope analysis of carbon and nitrogen and analysis of microdebris in dental calculus. Dietary intake was explored over short and long timescales. Bulk bone collagen was analysed from humans from the Queen’s Chapel of the Savoy (QCS) (*n* = 66) and the St Barnabas/St Mary Abbots (SB) (*n* = 25). Incremental dentine analysis was performed on the second molar of individual QCS1123 to explore childhood dietary intake. Bulk hair samples (*n* = 4) were sampled from adults from QCS, and dental calculus was analysed from four other individuals using microscopy. In addition, bone collagen from a total of 46 animals from QCS (*n* = 11) and the additional site of Prescot Street (*n* = 35) was analysed, providing the first animal dietary baseline for post-medieval London. Overall, isotopic results suggest a largely C_3_-based terrestrial diet for both populations, with the exception of QCS1123 who exhibited values consistent with the consumption of C_4_ food sources throughout childhood and adulthood. The differences exhibited in δ^15^N_coll_ across both populations likely reflect variations in diet due to social class and occupation, with individuals from SB likely representing wealthier individuals consuming larger quantities of animal and marine fish protein. Microdebris analysis results were limited but indicate the consumption of domestic cereals. This paper demonstrates the utility of a multidisciplinary approach to investigate diet across long and short timescales to further our understanding of variations in social status and mobility.

**Electronic supplementary material:**

The online version of this article (10.1007/s12520-019-00910-8) contains supplementary material, which is available to authorized users.

## Introduction

Between the seventeenth and nineteenth centuries, Britain witnessed industrialisation and urbanisation on an unprecedented scale. The expanding reach of the British Empire and successive conflicts, such as the Napoleonic Wars (1793–1815), saw Britain emerge as one of the world’s most powerful trading nations. Trade routes linked Britain to the Americas, Africa and Asia and opened the door for imports, such as tea, maize and sugarcane (Mintz 1985; Thirsk 2007). New transport networks facilitated the movement of people, goods and animals across the country, and important agricultural developments increased both the yield and variety of crops produced (Drummond and Wilbraham 1969; Williamson 2002). During this period, Britain stood at the forefront of these agricultural developments, and its capital city rapidly became an established centre for global trade and commerce. London, therefore, provides an ideal setting to examine dietary habits during this transformative period of history. While written documents attest to the changing nature of diets during this time, bioarchaeological data has the potential to complement or challenge these assumptions by providing insights at the population- or individual-level.

Increased urban development in Britain over the past decade has resulted in a growing number of rescue excavations of post-medieval cemeteries and crypts across the country, and in particular in London (Molleson et al. 1993; Cowie et al. 2008; Sibun and Ponce 2018). Post-medieval contexts often present favourable preservation conditions for human remains facilitating biomolecular analysis which, in conjunction with historical and archaeological datasets, can provide detailed insights into the lives of past populations. Furthermore, the preservation of different tissues, such as hair and bone, has enabled the investigation of diet over both short and long timescales, respectively (Richards 2006; Wilson and Cadwallader 2010; Beaumont et al. 2013a; Brown and Alexander 2016). Carbon and nitrogen stable isotope analysis is an established method for palaeodietary reconstruction and is increasingly being applied to post-medieval populations from Britain (Müldner and Richards 2005, 2007a; Nitsch et al. 2010, 2011; Roberts et al. 2012; Beaumont et al. 2013a; Brown and Alexander 2016) and northern parts of Europe (Jørkov and Gröcke 2016; Holder et al. 2017). Research on London populations has included the analysis of higher status individuals (Trickett 2006), middle to working class populations (Molleson et al. 1993; Nitsch et al. 2010, 2011) and military populations (Roberts et al. 2012).

This research examines diet across the social spectrum, extending the current isotopic dataset for post-medieval London to two new sites with differing histories. Significantly, we have also analysed animals from the city, creating the first animal baseline for London for this period which enables a better understanding of both dietary signatures for the city and animal husbandry practices. Carbon (δ^13^C) and nitrogen (δ^15^N) stable isotope analyses of human bone collagen and hair keratin are used to explore dietary variation among and between the people buried at Queen’s Chapel of the Savoy (c. AD 1510 to 1854) and St Barnabas/St Mary Abbots (c. AD 1831 to 1853). In addition, dental calculus samples from four Queen’s Chapel of the Savoy individuals were analysed for food-related and non-dietary debris using microfossil analyses. The overall burial population at QCS includes civilians, hospital patients, prisoners and military personnel (Savoy Chapel Burial Records 1680–1854; Sibun and Ponce 2018). The site of St Barnabas/St Mary Abbots provides a comparison with individuals interred within the relatively affluent district of Kensington (Cathcart-Borer 1973; Croot 2004).

## Diet in post-medieval Britain

Post-medieval London was socially stratified with pronounced differences between the diet and health of varying social classes, as shown by Charles Booth’s Poverty Maps of London (1898–1899), historical accounts of post-medieval diet (Mayhew 1861; Smith 1864) and osteological findings of increased mortality and nutritional disease in children of a lower socioeconomic status (Pinhasi et al. 2006; DeWitte et al. 2016). Fluctuations in food availability, inflated food prices and a decline in the demand for labour left many of the working class facing poverty (Mokyr 1988). In contrast, the wealthiest members of society could supplement their diet with luxury imported foods (Drummond and Wilbraham 1969). The amount of animal protein consumed also varied according to class (Olsen 1999). The elite could afford quality sources of animal protein and fish (Dyer 1988; Freeman 1989; Thirsk 2007) whereas the poorest members of society were restricted to cheap offcuts and items, such as smoked herring (Thirsk 2007; Clayton and Rowbotham 2009). In this study, nitrogen stable isotope analysis was used to investigate variations in animal protein consumption, although it is not possible to distinguish between meat or secondary animal products (O’Connell and Hedges 2001; Privat et al. 2005).

During the seventeenth century, the Savoy Hospital served as a military infirmary, accepting injured soldiers and seamen from the English Civil War (1642–1651) and Anglo-Dutch Wars (1652–1667) (Firth 1902; Keevil 1957). The military personnel buried in the Queen’s Chapel of the Savoy cemetery would therefore have been allocated weekly rations during their years of service which is well-documented (The Privy Council, Great Britain 1757; MacDonald 2014). The New Model Army (1645–1660) was supplied with rations of bread, cheese and salted meats (Gentles 1992; Nusbacher 2000; MacDonald 2014), and, in later years, naval personnel were given 4 lb of beef and 2 lb of pork a week (The Privy Council, Great Britain 1757). In the Georgian era, salted fish was dropped from the provision list, but ships were supplied with fishing equipment enabling the crew to access marine food sources (Vale 2008). The inclusion of both military personnel and civilians in the burial population at the Queen’s Chapel of the Savoy means that some variation in protein intake would be expected. In contrast, individuals from St Barnabas/St Mary Abbots may have been able to access larger quantities of protein and afford more expensive cuts of meat and fish due to their higher socioeconomic status (Oddy 1970; Greaves 2018).

The vast majority of the post-medieval British diet derived from C_3_ cereals, with the poor eating inexpensive rye or barley bread and the upper classes consuming wheat bread (Mitchell 1996; Thirsk 2007). The contribution of C_4_ cultigens (e.g. sugarcane, millet, maize) was more limited; however, there was a sharp increase in the consumption of sugar due to the colonization of the West Indies and abolition of the Sugar Tax in 1874 (Drummond and Wilbraham 1969; Rowbotham and Clayton 2008; Mant and Roberts 2015). The growing popularity of sugar was an important dietary transition, but as bone collagen carbon is derived mainly from dietary protein, the contribution of sugarcane (a carbohydrate) is less visible using the methods applied in this study (Ambrose and Norr 1993; Ambrose et al. 1997). Furthermore, sugar did not become an established component of the British diet across all classes until the 1880s (Deerr 1950) and burials at QCS and SB both ended around 1850. Maize was introduced to Britain from the Americas but took a long time to gain popularity (Greig 1996; Schmidt et al. 2005); however, its use as a famine food during the Great Irish Famine (1845–52) has been identified using stable isotope analysis (Beaumont and Montgomery 2016).

## Dietary reconstruction using stable isotope analysis

Stable light isotope analysis is a widely applied methodology in bioarchaeology (Schoeninger and Moore 1992; Jaouen and Pons 2016). When the body is not experiencing nutritional stress, the carbon and nitrogen isotopic values of bone collagen largely reflect the protein component of the diet up to 10–30 years prior to death (Hedges et al. 2007). Hair keratin also reflects dietary protein but grows incrementally at an average of 1 cm per month and offers considerable potential in terms of diachronic information (Wilson et al. 2007). Although largely under-represented in the archaeological record, long lengths of hair have been used to interpret changes in diet, health and mobility (Wilson et al. 2007; Thompson et al. 2014; D’Ortenzio et al. 2015). Tooth dentine also forms incrementally during tooth formation, and, unlike bone, it does not undergo remodelling. Teeth therefore provide dietary information during childhood and adolescence, depending on the timing of tooth development (Eerkens et al. 2011).

Carbon isotope ratios can be used to distinguish between different plant groups based on their photosynthetic pathways. Most temperate plants use the C_3_ photosynthetic pathway, whereas tropical grasses (e.g. maize, sugarcane) use the C_4_ pathway (Calvin and Benson 1948; Hatch and Slack 1966). These pathways lead to distinctive isotopic values with C_3_ plants exhibiting δ^13^C values between − 19 and − 35‰ and C_4_ plants typically exhibiting values between − 8 and − 13‰ (O’Leary 1981; Ambrose 1990). Due to the diet-tissue offset in δ^13^C, the value generated from the bone collagen of an individual with a complete C_3_ protein diet would average around − 22‰, whereas a 100% C_4_ diet would be − 8‰ (Kellner and Schoeninger 2007). Carbon isotope values also differ between marine species and C_3_ terrestrial-based resources because CO_2_ in the ocean is derived from dissolved inorganic carbon and enriched with respect to atmospheric CO_2_ (Schwarcz and Schoeninger 2011; Eriksson 2013). Therefore, marine flora and fauna have elevated δ^13^C values in comparison to terrestrial ones (Schwarcz and Schoeninger 2011; Eriksson 2013). Nitrogen isotopes can provide information on trophic level due to a step-wise fractionation of ^15^N enrichment as the food chain progresses, leading to a rise in δ^15^N values by around 2–5‰ with each trophic step (Bocherens and Drucker 2003; O’Connell et al. 2012). There is also a slight enrichment of 0–2‰ in δ^13^C as trophic levels increase (Bocherens and Drucker 2003). As aquatic food chains tend to be longer than terrestrial ones, animals feeding exclusively on marine sources display δ^15^N values around 9‰ more positive than those fed on terrestrial sources (Schoeninger et al. 1983; Schoeninger and DeNiro 1984).

Precise dietary reconstruction using human bone collagen is challenging as many factors can result in enriched nitrogen values in plants and their consumers, including environmental conditions, manuring and physiological stress (Bogaard et al. 2007; Katzenberg and Lovell 1999; Schwarcz et al. 1999; Britton et al. 2008; Fraser et al. 2011; Hertz et al. 2015). However, by combining multiple measurements of different isotopes and tissues, interpretations of dietary composition (Webb et al. 2013; Jørkov and Gröcke 2016), weaning practices (Henderson et al. 2014; Tsutaya and Yoneda 2015), nutritional stress (Hobson et al. 1993; Beaumont et al. 2015) and disease (Reitsema 2013) are possible. Furthermore, analysis of bone collagen from local animals is required to provide baselines against which human dietary signals can be examined.

### Exploring food consumption through the analysis of dental calculus

Dental calculus (calcified dental plaque) can also provide dietary information for an individual. As plaque mineralises in the mouth, microdebris and biomolecules originating from the oral microbiome, dietary sources and environmental microparticles can become trapped in the matrix (Armitage 1975; Dobney and Brothwell 1988; Warinner et al. 2015; Weyrich et al. 2015). Dental calculus can offer unique insights into food consumption and culinary practices through the identification of ancient proteins, such as milk (Warinner et al. 2014) and plant proteins (Hendy et al. 2018), as well as through the retrieval of plant microfossils (Dudgeon and Tromp 2014; Buckley et al. 2014). Furthermore, dental calculus can also entrap non-dietary debris relating to environmental pollutants, craftwork and oral hygiene activities (Hardy et al. 2016; Radini et al. 2017). To gain a snapshot of diet at the individual-level, analysis of calculus microdebris was performed on four individuals from the Queen’s Chapel of the Savoy. This method has previously been applied to urban medieval populations from the UK (Radini et al. 2016) and Europe (Lazzati et al. 2016), and it is becoming a complementary line of evidence for isotope studies (Wang et al. 2016; Baldoni et al. 2018).

## Materials and methods

**Fig. 1:**
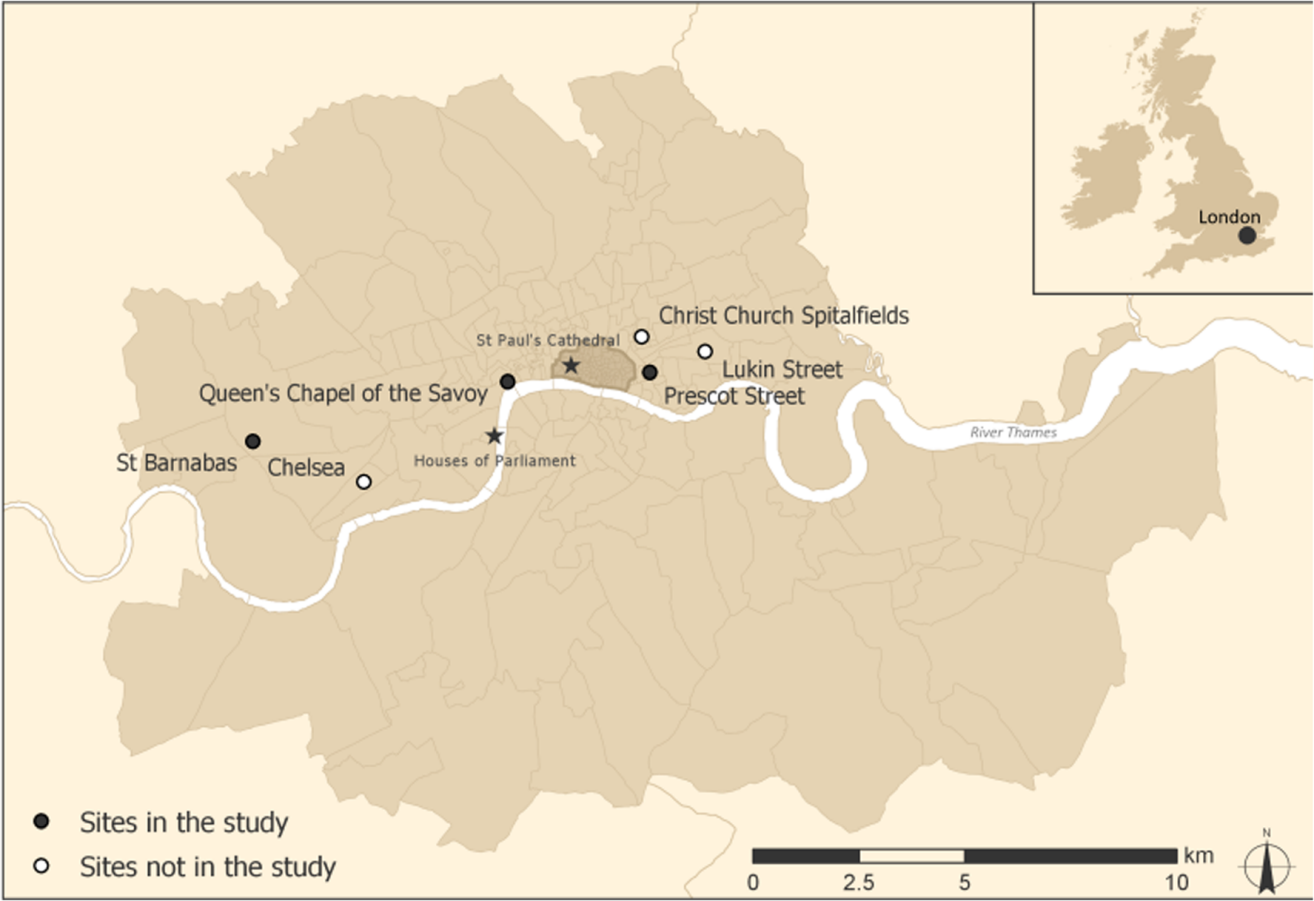
Map of Victorian London showing the locations of the sites of study, post-medieval sites mentioned in the text and landmarks. The city of London is shaded (map by Helen Goodchild, Department of Archaeology, Univeristy of York; 1851 parish data from Satchell et al. (2016)

Human and animal samples for this research were derived from the following three sites across London: the Queen’s Chapel of the Savoy, St Barnabas and Prescot Street (Fig. 1).

### The Queen’s Chapel of the Savoy

The Queen’s Chapel of the Savoy (QCS) cemetery is located approximately 150 m northwest of Waterloo Bridge, in the City of Westminster. The site was excavated by the Archaeology South-East, UCL, in 2011 in response to the redevelopment of the chapel, and 609 burials were excavated (Sibun and Ponce 2018). Historical and burial records indicate that the cemetery population represents a range of individuals, including parishioners, hospital patients, military personnel and criminals (Savoy Chapel Burial Records 1680–1854; Somerville 1960). The cemetery was first used in 1523 when a hospital for the poor was built in accordance with the wishes of the late King Henry VII (Thornbury 1878a). In addition to the hospital, the precinct included the Queen’s Chapel (formerly St John’s Chapel) and churchyard which served as a burial ground for the parishioners (Somerville 1960). During the English Civil War (1642–1651), the Savoy Hospital was converted to a military infirmary and later accepted injured seamen from the first and second Anglo-Dutch Wars (1652–1667) (Firth 1902; Keevil 1957). In 1679, the site underwent another transformation when part of the precinct became the barracks for the Foot Guards and a prison (Strype 1720). The hospital was dissolved in 1702, and, by 1816, all but the chapel had been demolished (Somerville 1960). Throughout the site's many transformations, the Queen's Chapel of the Savoy churchyard remained in use until 1854 when the cemetery was closed in accordance with the 1853 Burial Act (Burial Act 1853). Given the continual use of the site over 300 years, the density of graves and level of truncation, it was not possible to phase the burials. However, it is most likely that the surviving graves represent the later years of use (Sibun and Ponce 2018).

The use of QCS as a hospital and military infirmary is supported by the material culture and osteological evidence (Sibun and Ponce 2018). Of the total buried population, 85% (*n* = 519) were adults age 18 + years of age and the remaining 15% (*n* = 90) were sub-adults (foetus to 17 years old). Of the adults for whom sex could be estimated, 76% were male (325/430) and 24% female (105/430). The imbalance between the adult and sub-adult population and between the sexes supports the use of the burial ground for military personnel. In the overall skeletal population, 68% (417/609) individuals were affected by some form of trauma, including fractures of the upper and lower limbs, the skull, and two examples of cranial gunshots. Furthermore, 25% (154/609) of all individuals showed osteological evidence of infection. Records from Charles Hayes, a surgeon at the Savoy Hospital in the eighteenth century, indicate that sexual transmitted diseases, such as gonorrhoea and syphilis, were common among the soldiers at QCS (Hales 1770). Recorded finds including a musket ball, 138 fragments of clay pipes and dress accessories further attest to the use of the site during the seventeenth–eighteenth centuries. The total number of finds from the site was relatively low, but these finds, along with a small number of faunal remains, are consistent with rubbish disposal. While the faunal material cannot be directly linked to the human burials, it is unlikely to be intrusive, as pottery finds indicate that the rate of rubbish disposal slowed down in the eighteenth century, and, following the disuse of the cemetery, the ground remained undisturbed (Sibun and Ponce 2018).

### St Barnabas/St Mary Abbots

St Barnabas church is located in West Kensington, London, on Addison Road. In 1829, it was consecrated and designated a chapel of ease to St Mary Abbots, which is located approximately 1 mile east of the site. Records indicate that the sample population was originally interred at St Mary Abbots between 1831 and 1853 before being reburied at St Barnabas, although the precise date of the transfer is unknown (Goldsmith 2016). For the purpose of this study, the population will be referred to as St Barnabas (SB). In 1991, individuals were exhumed from the crypt in response to construction work, and a selection of individuals was retained for further study (Goodyear et al. 1994). It is likely that some individuals represent lifelong residents of the area. In the medieval period, Chelsea and Kensington were an attractive retreat for the upper classes and nobility, with King Henry VIII acquiring the manor of Chelsea in 1536 (Cathcart-Borer 1973; Croot 2004). The construction of the Royal hospital in 1682 drew more people to settle in the area. By the seventeenth and eighteenth centuries, Kensington became increasingly urbanised, and St Mary Abbots was extended to meet the demands of the growing congregation (Hobhouse 1986; Cowie et al. 2008).

### Prescot Street

The QCS cemetery contained few animal bones, and the site’s complex urban stratigraphy means that the animal remains recovered may not be directly associated with the human burials. Therefore, in addition to 11 animal remains from QCS, a larger comparative post-medieval faunal assemblage was also analysed from Prescot Street (PS), East London, to provide an isotope baseline. Prescot Street was excavated by LP Archaeology in 2006. In the early medieval period, the land was used for agriculture and the disposal of domestic refuse (Richardson 2010). Towards the end of the fifteenth century, Aldgate developed into an affluent residential area, and Prescot Street itself was built in 1678. The area underwent significant development in the eighteenth–nineteenth centuries due to the increased demand for housing in London which resulted in the construction of densely spaced lower-quality housing. The faunal remains analysed in this study were recovered from quarry pits from the 16^th^ to 19^th^ centuries (Reilly 2014).

### Isotope analysis of human and faunal remains

Ribs were preferentially sampled from human burials; however, other elements were sampled when these were unavailable (Table 1). From QCS, 66 adults were selected for bone collagen, including 26 females and 40 males. Four additional adult individuals were sampled for bulk hair keratin analysis. Animal bones (*n* = 11) were also sampled from QCS, excavated from cemetery deposits. A total of 25 adults were sampled from SB for bone collagen, 12 females and 13 males. Among these individuals, one female, SB48, had scalp hair. The results of incremental hair analyses have already been published for this individual (Brown and Alexander 2016), and, for this study, bulk values were calculated for comparison. Animal remains representing a range of species were also sampled from Prescot Street (*n* = 35). No attempt was made to distinguish between sheep and goat bones.

**Table 1:** δ^13^C_coll_ and δ^15^N_coll_ values for humans from QCS and SB

Site	Sample	Element	Sex	Age (yrs)	δ^13^C_coll_ ‰VPDB	%C	δ^15^N_coll_ ‰AIR	%N	C/N	Collagen yield %
Queen’s Chapel of the Savoy	QCS116	Rib	F	18–30	− 19.0	42.6	12.6	15.7	3.2	1.3
	QCS117	Skull	M?	18 +	− 19.9	45.5	12.8	16.8	3.2	11.3
	QCS117b	Skull	M?	18 +	− 19.4	42.7	12.8	15.5	3.2	2.6
	QCS121	Rib	F	18 +	− 19.4	43.0	12.2	15.9	3.2	14.7
	QCS122	Rib	F	18–30	− 19.2	41.2	10.1	15.3	3.1	12.2
	QCS123	Rib	M	45 +	− 18.3	44.8	13.2	16.4	3.2	13.3
	QCS124	Rib	F?	45 +	− 18.7	41.5	12.5	15.4	3.2	11.0
	QCS147	Rib	M	31–45	− 19.7	36.3	12.1	13.1	3.2	2.7
	QCS163	Rib	F	18 +	− 19.2	36.0	11.3	12.9	3.3	2.2
	QCS208	Mandible	M	18 +	− 20.0	37.6	11.1	13.7	3.2	4.8
	QCS268	Rib	F	18–30	− 19.6	43.9	13.7	16.2	3.2	12.2
	QCS306	Rib	M	45 +	− 19.6	44.0	13.2	16.3	3.2	16.9
	QCS338	Rib	M?	18 +	− 20.0	43.7	12.7	16.2	3.2	12.5
	QCS365	Rib	M	31–45	− 21.1	26.9	9.9	9.7	3.2	1.6
	QCS427	Rib	F	31–45	− 19.6	44.1	12.8	16.3	3.2	15.8
	QCS448	Rib	M?	45 +	− 19.5	44.5	12.2	16.2	3.2	12.3
	QCS490	Rib	F	31–45	− 19.3	44.8	13.0	16.5	3.2	2.2
	QCS495	Rib	F	31–45	− 17.6	44.5	12.2	16.5	3.1	9.7
	QCS534	Rib	F	45 +	− 19.1	42.9	12.4	15.7	3.2	5.9
	QCS566	Rib	F	31–45	− 19.5	43.5	13.0	15.9	3.2	13.1
	QCS589	Rib	M	45 +	− 19.0	44.4	13.6	16.2	3.2	5.9
	QCS639	Rib	M	31–45	− 19.4	44.3	11.8	16.4	3.2	15.2
	QCS643	Rib	M	45 +	− 18.9	39.5	12.5	14.6	3.2	5.8
	QCS648	Skull	M	18–30	− 18.7	41.0	12.3	15.2	3.1	8.0
	QCS649	Rib	F	31–45	− 19.7	42.7	12.1	15.6	3.2	2.3
	QCS700	Rib	M	31–45	− 19.5	43.7	12.3	16.1	3.2	3.2
	QCS719	Rib	M	45 +	− 19.6	42.1	12.0	15.5	3.2	5.2
	QCS731	Humerus	M	45 +	− 18.6	43.1	13.8	15.8	3.2	15.1
	QCS735	Rib	M	31–45	− 19.6	42.6	13.3	15.4	3.2	11.9
	QCS751	Rib	M	31–45	− 19.9	42.8	11.7	15.9	3.2	13.6
	QCS801	Rib	M	31–45	− 19.5	43.9	12.2	16.1	3.2	12.4
	QCS813	Rib	M	18–30	− 20.0	37.3	10.5	13.2	3.2	3.4
	QCS930	Rib	M	45 +	− 19.7	43.7	12.5	16.1	3.2	15.5
	QCS939	Rib	M	18 +	− 18.9	37.1	13.1	13.7	3.2	7.2
	QCS996	Rib	M	31–45	− 19.0	43.9	12.5	16.2	3.2	10.9
	QCS1012	Rib	M	18–30	− 19.6	43.0	11.4	15.8	3.2	1.8
	QCS1020	Rib	F	18–30	− 19.4	44.6	12.2	16.5	3.2	6.2
	QCS1090	Tibia	M?	18–30	− 20.2	43.1	11.4	15.7	3.2	1.3
	QCS1123	Rib	M	31–45	− 13.0	43.8	10.8	16.2	3.2	15.1
	QCS1138	Rib	F	45 +	− 19.6	45.2	11.3	16.7	3.2	14.8
	QCS1150	Rib	F	31–45	− 20.1	45.5	9.9	16.9	3.1	7.2
	QCS1249	Rib	M	31–45	− 20.0	43.5	10.9	16.0	3.2	9.7
	QCS1262	Rib	M	31–45	− 18.8	43.6	11.1	15.9	3.2	12.2
	QCS1289	Rib	M	18–30	− 19.4	42.2	12.0	15.2	3.2	3.4
	QCS1304	Rib	F?	18–30	− 20.1	43.8	11.9	16.3	3.1	8.5
	QCS1360	Rib	F	18–30	− 20.2	44.2	10.2	15.9	3.3	11.1
	QCS1369	Rib	M	45 +	− 19.7	44.0	11.7	16.1	3.2	20.9
	QCS1415	Rib	M	18–30	− 19.6	44.3	12.1	16.5	3.1	12.0
	QCS1424	Rib	F?	18–30	− 19.6	44.4	12.3	16.5	3.1	7.7
	QCS1498	Rib	M	45 +	− 19.9	45.7	13.1	16.8	3.2	3.7
	QCS1518	Rib	F	18 +	− 19.3	44.0	12.5	15.9	3.2	17.8
	QCS1558	Rib	M?	18–30	− 19.6	43.7	12.6	16.0	3.2	0.9
	QCS1667	Rib	F	45 +	− 19.3	44.5	12.5	16.3	3.2	4.0
	QCS1736	Rib	M	31–45	− 19.9	43.5	11.6	16.1	3.2	14.7
	QCS1746	Rib	M	18–30	− 19.2	33.0	11.7	12.2	3.1	4.2
	QCS1767	Rib	F?	45 +	− 19.7	45.6	12.6	16.8	3.2	11.3
	QCS1792	Tibia	F?	45 +	− 19.4	43.2	12.3	15.9	3.2	4.9
	QCS1804	Rib	F	31–45	− 18.8	43.7	13.2	16.1	3.2	15.7
	QCS1810	Rib	F	45 +	− 19.0	43.2	12.3	15.9	3.2	5.7
	QCS1817	Rib	F	45 +	− 18.8	43.3	12.8	15.9	3.2	13.3
	QCS1922	Rib	M?	31–45	− 19.8	43.3	11.0	15.7	3.2	2.4
	QCS1943	Rib	M	31–45	− 19.6	45.2	12.0	16.7	3.2	11.9
	QCS1961	Rib	M	18 +	− 19.4	32.6	12.3	12.0	3.2	3.2
	QCS1968	Rib	F	18–30	− 19.7	44.2	12.2	16.3	3.2	13.9
	QCS1981	Calcaneus	M	31–45	− 19.8	45.3	11.9	16.6	3.2	8.8
	QCS1998	Rib	M?	31–45	− 19.0	45.5	14.4	16.7	3.2	13.0
St Barnabas	SB07	Mandible	F	45 +	− 19.6	38.0	12.6	13.9	3.2	7.5
	SB08	Mandible	M	45 +	− 19.8	42.0	13.3	15.3	3.2	1.3
	SB09	Rib	F	45 +	− 19.3	41.5	12.7	15.0	3.3	9.4
	SB10	Rib	M	45 +	− 19.0	43.3	14.0	14.6	3.3	2.5
	SB11	Rib	M	45 +	− 19.2	43.5	13.5	15.4	3.3	2.2
	SB12	Rib	M	45 +	− 19.3	43.7	13.2	15.2	3.3	5.3
	SB15	Calcaneus	M	45 +	− 19.3	43.6	13.8	15.4	3.3	10.8
	SB16	Rib	F	45 +	− 18.3	44.1	13.2	15.8	3.3	3.7
	SB17	Rib	M	45 +	− 18.3	45.6	12.6	16.5	3.3	8.0
	SB18	Rib	M	45 +	− 18.1	48.2	14.6	17.0	3.3	8.3
	SB21	Rib	M	45 +	− 19.3	44.6	13.8	15.9	3.3	11.3
	SB26	Rib	F	45 +	− 19.2	43.5	12.5	15.8	3.3	9.4
	SB27	Rib	F	45 +	− 19.8	43.7	11.9	16.0	3.2	5.4
	SB30	Rib	M	18–30	− 19.0	44.6	13.7	16.0	3.3	14.7
	SB34	Rib	M	45 +	− 18.4	42.7	13.9	15.4	3.3	11.9
	SB36	Rib	M	18 +	− 17.8	43.1	14.2	15.9	3.3	9.4
	SB43	Rib	F	45 +	− 19.1	42.8	13.6	15.5	3.3	11.1
	SB44	Rib	F	45 +	− 18.1	44.8	15.2	15.8	3.3	12.4
	SB45	Rib	F	18–30	− 19.6	45.9	13.4	16.4	3.3	11.7
	SB46	Rib	F	31–45	− 19.6	43.1	13.5	15.8	3.2	5.8
	SB48	Rib	F	45 +	− 18.1	40.9	13.6	14.9	3.2	8.9
	SB53	Rib	M	31–45	− 19.7	42.9	12.9	15.6	3.3	12.0
	SB54	Rib	F	45 +	− 19.7	44.7	13.5	16.2	3.3	9.2
	SB57	Rib	F	45 +	− 20.3	43.7	13.9	14.9	3.4	10.1
	SB58	Rib	M	45 +	− 18.5	41.2	14.0	14.8	3.2	2.6

Bone collagen was extracted using a modified Longin (1971) method with additional ultrafiltration (Brown et al. 1988; Richards and Hedges 1999). Bone samples (300–400 mg) were demineralised by immersion in 0.6 M HCl for 1–4 days. The resultant pseudomorphs were rinsed three times in distilled water, and the residue was gelatinized in pH 3 HCl at 80 °C for 48 h. The soluble collagen solution was filtered to remove insoluble residues (Brock et al. 2013), and the supernatants were then ultrafiltered to isolate the high molecular weight > 30 kDa fraction and lyophilized in a freeze drier. Serial sectioning of dentine of the lower second molar of individual QCS1123 followed Beaumont et al.’s (2013b) method 2. The M2 was cleaned with a sandblaster, and the crown was cut in half leaving two halves with one root each. As much of the enamel as possible was removed from one half using a hand-held drill. The full longitudinal root section from the tip of the crown to the bottom of the root was demineralised and sectioned into 1 mm increments using a scalpel. Demineralization took around three weeks. Each 1 mm section was gelatinized and freeze-dried without filtration.

For each individual or individual tooth section, approximately 1.0 mg of the resulting purified collagen was weighed in duplicate into tin capsules (8 × 5 mm, Elemental Microanalysis, UK) for analysis. A control sample of modern homogenised bovine bone was weighed and processed with each batch of the samples. The isotopic and collagen composition of the control was previously determined; so, it served as a quality control of sample processing (Budzikiewicz and Grigsby 2006).

The human hair samples from QCS were assessed for surface condition at the University of Bradford Analytical Centre using an FEI Quanta 400 Environmental Scanning Electron Microscope. Hair samples were prepared for sampling according to standard protocols (Thompson et al. 2014). Adherent soil and exogenous organic deposits were removed from the surface by overnight soaking/gentle agitation in 2:1 (vol/vol) methanol/chloroform, followed by sonication within scintillation vials in an ultrasonication bath (3 × 15 min). The organic solvent was then removed, and the hair sample was rinsed in deionized water (3 separate washes, each with sonication). The final wash was decanted off, and the cleaned sample was frozen, lyophilized and preconditioned for weighing into tin capsules.

The δ^13^C and δ^15^N ratios of the QCS, SB and PS bone collagen were measured using a Secron continuous-flow 20–22 isotope ratio mass spectrometer (CF-IRMS) interfaced with a Universal Sercon GSL preparation at BioArCh, University of York. The QCS hair samples were analysed by EA-IRMS using a ThermoFinnigan FlashEA 1112 elemental analyser coupled to a DeltaPlus XL multicollector mass spectrometer at the University of Bradford Isotope Laboratory. When sufficient material was available, the hair samples were measured in duplicate. The condition of the QCS hair samples meant that only bulk measurements were possible for this study.

All isotopic values are reported as the ratio of the heavier isotope to the lighter isotope (^13^C/^12^C or ^15^N/^14^N) as δ values in parts per mille (‰) relative to international standards, VPDB for δ^13^C and atmospheric N2 (AIR) for δ^15^N, using the following equation: [δ = (*R*_sample_ − *R*_standard_) / *R*_standard_] (Coplen 1994). At the University of York, in-house fish gelatine standards were calibrated to international reference materials IAEA-N-2 (ammonium sulphate, δ^15^N_AIR_ = +  20.30‰), IAEA-600 (caffeine, δ^15^N_AIR_ = + 1.0‰, δ_13_C_VPDB_ − 27.77‰) and IA-R006 (sugar cane δ^13^C_VPDB_ − 11.64‰). At the University of Bradford, the following reference materials were used: IAEA-600 (caffeine, δ_15_N_AIR_ = + 1.0‰, δ^13^C_VPDB_ − 27.77‰), IAEA-N-2 (ammonium sulphate, δ^15^N_AIR_ = + 20.30‰) and IAEA-CH-3 (cellulose, δ^13^C_VPDB_ − 24.72‰). In addition, an in-house fish gelatine and bovine liver standard was used. Measurement reproducibility was ± 0.3‰ or better for both δ^13^C_coll_ and δ^15^N_coll_.

### Microdebris analysis of QCS human dental calculus

For the purpose of this study, it was possible to analyse samples of dental calculus from the following four individuals from the Queen’s Chapel of the Savoy: QCS427, QCS819, QCS1746 and QCS1961. The calculus samples were removed from different teeth but all originated on the lingual surface. Following the established protocols (Cristiani et al. 2018), surface contaminants were removed by brushing the surface of the sample with an acupuncture needle used in conjunction with a 0.06 M solution of HCl. The samples clean weight varied between 6 and 8.5 mg (QCS427: 6 mg; QCS819: 6.5 mg; QCS1746 8.5 mg; QCS1961: 7 mg). Once free of any visible contaminants, the calculus was placed in sterile Eppendorf tubes, rinsed in ultrapure water, transferred to new Eppendorf tubes and demineralised in a 0.06 M solution of HCl. The demineralised calculus, often in small flecks, was siphoned out of the tubes using an Eppendorf pipette and placed onto a sterile glass slide. A drop of a 50:50 glycerol and ultrapure water solution was added to the sample before adding a coverslip and sealing the slide. The slides were scanned in their entirety using an Olympus inverted light microscope under magnification between 400 and 630× and complemented by observation under polarised light. The identification of retrieved microfossils was based on anatomical and optical properties and through comparison with a built for the purpose reference collection hosted at the University of York (Warinner et al. 2014; Radini et al. 2016).

## Results

### Faunal and human bone collagen

**Fig. 2:**
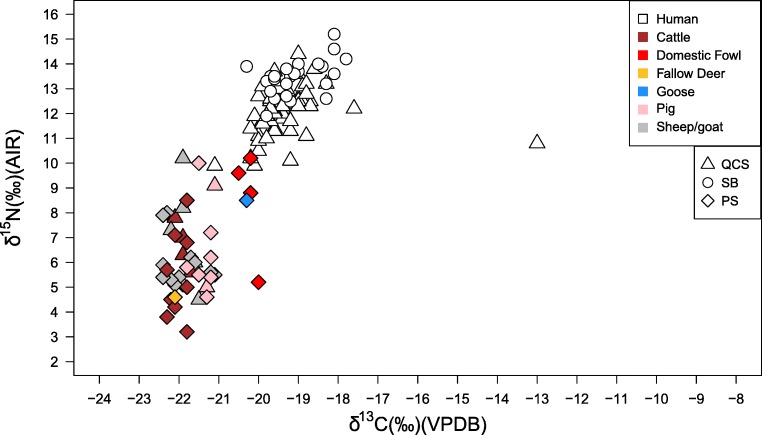
Human and faunal bulk collagen results from QCS, SB and PS

The QCS and SB human bone collagen results are displayed in Table 1 with faunal results from QCS and PS presented in Table 2. The bone collagen results for all sites are plotted in Fig. 2, and summary statistics is presented in Table 3. Samples were subjected to a series of quality controls to ensure the accuracy of the data and assess preservation, and these included a collagen yield of 1% or higher, C/N ratio of 2.9–3.6, %C of ca.15–48% and %N of ca. 5–17% (DeNiro 1985; Ambrose 1993, Ambrose 1990; van Klinken 1999; Sealy et al. 2014). Sample QCS1558 had a collagen yield of 0.9% but met all other criteria, indicating good sample quality and was therefore carried forward for analysis.

**Table 2:** δ^13^C_coll_ and δ^15^N_coll_ values for fauna from QCS and PS

Site	Sample	Species	Element	δ^13^C_coll_ ‰VPDB	%C	δ^15^N_coll_ ‰AIR	%N	C/N	Collagen yield%
Queen’s Chapel of the Savoy	QCS101C1	*Bos taurus*	1st Phalanx	− 22.1	44.3	7.8	16.2	3.2	5.6
	QCS104C1	*Bos taurus*	1st Phalanx	− 21.9	44.2	6.3	16.4	3.1	4.4
	QCS104C2	*Bos taurus*	1st Phalanx	− 21.9	43.5	7.0	16.0	3.2	5.0
	QCS117C1	*Bos taurus*	Astragalus	− 21.7	44.4	5.6	16.4	3.2	7.6
	QCS101S1	*Ovicaprid*	Metatarsal	− 21.5	44.7	4.5	16.6	3.2	7.0
	QCS101S2	*Ovicaprid*	Metatarsal	− 21.6	44.6	6.0	16.4	3.2	4.3
	QCS104S1	*Ovicaprid*	Metatarsal	− 21.9	35.6	8.2	12.6	3.3	2.1
	QCS117S1	*Ovicaprid*	Scapula	− 21.9	43.8	10.2	16.3	3.1	10.7
	QCS117S2	*Ovicaprid*	Scapula	− 22.2	45.0	7.3	16.8	3.1	7.6
	QCS117P1	*Sus*	4th Metatarsal	− 21.3	44.8	5.0	16.6	3.2	5.3
	QCS117P2	*Sus*	Tibia	− 21.1	44.4	9.1	16.6	3.1	9.9
Prescot Street	PCSG02	*Ovicaprid*	Scapula	− 21.9	41.4	5.1	15.4	3.1	16.7
	PCSG03	*Ovicaprid*	Mandible	− 21.1	43.2	5.5	15.8	3.2	14.1
	PCSG10	*Ovicaprid*	Metatarsal	− 22.0	42.7	5.4	15.8	3.2	15.9
	PCSG12	*Ovicaprid*	Metacarpal	− 22.1	43.2	5.1	16.1	3.1	18.7
	PCSG16	*Ovicaprid*	Humerus	− 21.2	44.1	5.6	16.1	3.2	9.2
	PCSG33	*Ovicaprid*	Scapula	− 22.2	43.8	5.3	16.3	3.2	20.3
	PCSG36	*Ovicaprid*	Mandible	− 21.7	41.9	6.2	15.3	3.2	17.6
	PCSG38	*Ovicaprid*	Humerus	− 22.3	32.0	8.0	11.5	3.2	5.6
	PCSG39	*Ovicaprid*	Femur	− 22.4	34.1	5.9	12.4	3.2	14.5
	PCSG40	*Ovicaprid*	Metacarpal	− 21.6	40.6	6.0	14.9	3.2	19.9
	PCSG45	*Ovicaprid*	Tibia	− 22.4	35.9	7.9	13.1	3.2	7.8
	PCSG46	*Ovicaprid*	Humerus	− 22.4	35.3	5.4	12.7	3.2	5.6
	PCC08	*Bos taurus*	Humerus	− 21.8	45.8	8.5	16.7	3.2	15.3
	PCC10	*Bos taurus*	Metacarpal	− 21.8	38.9	6.8	14.4	3.2	12.9
	PCC15	*Bos taurus*	Humerus	− 22.1	32.6	7.1	12.0	3.2	6.5
	PCC16	*Bos taurus*	Radius	− 22.3	37.4	3.8	14.0	3.1	12.5
	PCC32	*Bos taurus*	Radius	− 22.2	32.8	4.5	12.3	3.1	4.0
	PCC33	*Bos taurus*	Tibia	− 22.1	31.5	4.2	12.0	3.1	12.9
	PCC36	*Bos taurus*	Radius	− 22.3	31.2	5.7	11.6	3.1	7.0
	PCC37	*Bos taurus*	Metacarpal	− 21.8	42.8	5.0	15.7	3.2	12.3
	PCC38	*Bos taurus*	Humerus	− 21.8	36.1	3.2	13.9	3.0	5.4
	PCP03	*Sus*	Humerus	− 20.3	43.7	8.5	16.0	3.2	15.1
	PCP04	*Sus*	Mandible	− 21.5	37.6	10	13.7	3.2	12.8
	PCP07	*Sus*	Humerus	− 21.8	43.4	5.8	15.9	3.2	15.2
	PCP08	*Sus*	Mandible	− 21.2	40.4	5.4	14.8	3.2	25.8
	PCP09	*Sus*	Mandible	− 21.2	39.8	6.2	14.6	3.2	16.2
	PCP12	*Sus*	Mandible	− 21.2	30.7	7.2	11.6	3.1	7.4
	PCP13	*Sus*	Mandible	− 21.3	38.5	4.6	14.1	3.2	12.8
	PCP14	*Sus*	Phalanx	− 21.5	39.8	5.5	14.7	3.2	5.1
	PCD01	*Dama dama*	Metacarpal	− 22.1	42.7	4.6	15.7	3.2	15.2
	PCODF1	*Gallus*	Long bone	− 20.5	40.9	9.6	14.7	3.3	10.8
	PCODF2	*Gallus*	Long bone	− 20.0	40.2	5.2	14.8	3.2	13.3
	PCODF3	*Gallus*	Long bone	− 20.2	39.7	10.2	14.6	3.2	17.3
	PCODF4	*Gallus*	Long bone	− 20.2	42.2	8.8	15.3	3.2	20.2
	PCOG01	*Anser*	Long bone	− 20.3	43.7	8.5	16.0	3.2	5.8

**Table 3:** Summary isotopic data for QCS human and animal bone collagen, human bone collagen from SB and animal bone collagen from PS

			δ^13^C_coll_ ‰VPDB				δ^15^N_coll_ ‰AIR			
Species	Site	*n*	Mean	1 SD	Min	Max	Mean	1 SD	Min	Max
Human	QCS	66	− 19.4	0.9	− 21.1	− 13.0	12.2	0.9	9.9	14.4
	SB	25	− 19.1	0.7	− 20.3	− 17.8	13.5	0.7	11.9	15.2
*Bos taurus*	QCS	4	− 21.9	0.1	− 22.1	− 21.7	6.7	0.9	5.6	7.8
	PS	9	− 22.0	0.2	− 22.3	− 21.8	5.5	1.6	3.2	8.5
*Ovicaprid*	QCS	5	− 21.8	0.2	− 22.2	− 21.5	7.2	1.9	4.5	10.2
	PS	12	− 21.9	0.4	− 22.4	− 21.1	6.0	1.0	5.1	8.0
*Sus*	QCS	2	− 21.2	0.1	− 21.3	− 21.1	7.1	2.1	5.0	9.1
	PS	8	− 21.3	0.4	− 21.8	− 20.3	6.7	1.7	4.6	10.0
*Gallus*	PS	4	− 20.2	0.2	− 20.5	− 20.0	8.5	1.9	5.2	10.2
*Fallow deer*	PS	1	− 22.1	–	–	–	4.6	–	–	–
*Goose*	PS	1	− 20.3	–	–	–	8.5	–	–	–

The δ^13^C_coll_ values for sheep/goat and cows from both sites are consistent with expectations that ruminants in post-medieval Britain were raised on C_3_ fodder, but the QCS fauna on average are enriched in δ^15^N when compared to PS. The one exception for PS is sample PCC08 which produced a δ^15^N of 8.5‰, the maximum for all the cattle sampled across the sites of study. Furthermore, sample QCS117S1 (sheep/goat) produced a δ^15^N value of 10.2‰. Nitrogen values also reveal differences in the feeding regime of the animals at QCS and PS. Two pigs, QCS117P1 and QCS117P2, produced δ^15^N values 5.0‰ and 9.1‰, respectively. Similarly, one pig from PS (PCP13) gave a value of 4.6‰ and another (PCP04) produced a δ^15^N value of 10.0‰.

**Fig. 3:**
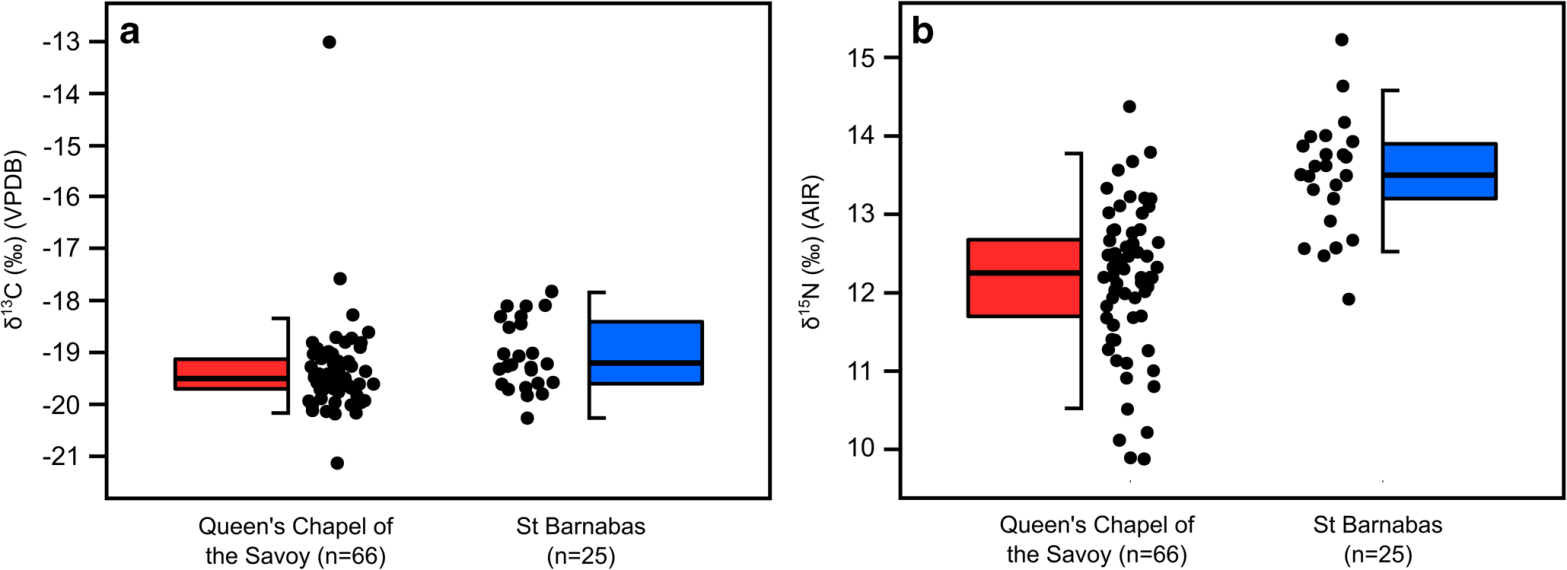
Boxplot comparison of the (**a**) δ^13^C_coll_ values for humans from QCS and SB and the (**b**) δ^15^N_coll_ values for humans from QCS and SB. The boxes indicate the inter-quartile range (IQR), Whiskers 1.5× the IQR and the black dots represent the result for each individual

Turning to human diets, the QCS population exhibits a wide range in δ^13^C_coll_ and δ^15^N_coll_ (8.1‰ and 4.5‰, respectively). For SB, the range for both isotopes is slightly smaller, at 2.5‰ for δ^13^C_coll_ and 3.3‰ for δ^15^N_coll_. Individual QCS1123 is an outlier with a δ^13^C_coll_ value of − 13.0‰, which is more than 2 standard deviations from the population mean (− 19.4‰) (Table 3). When comparing the two sites statistically (Mann–Whitney *U* test), significant differences were found between δ^13^C_coll_ (*U* = 569, *P* = < 0.03) and δ^15^N_coll_ (*U* = 185.5, *P* = < 0.001) values. Statistical tests were performed using SPSS Statistics Version 25. Comparing the mean bulk bone collagen values for QCS and SB (Fig. 3) reveals that the SB population is enriched in nitrogen. This may be reflective of differences in social status as St Barnabas and St Mary Abbots were located in an affluent area and local individuals may have consumed more animal and freshwater or marine protein than the average London civilian (Thirsk 2007).

Results for the faunal remains from both sites were pooled due to the highly variable δ^13^C_coll_ and δ^15^N_coll_ observed across both assemblages. In addition, the complex urban stratigraphy at QCS and depositional context for the animal remains mean that they may not directly relate to the diets of the burial population. Comparison of the QCS mean for humans and mean for all animals (− 21.6‰ for δ^13^C_coll_ and 6.5‰ for δ^15^N_coll_) reveals that humans are on average 2.2‰ higher for δ^13^C_coll_ and 5.7‰ for δ^15^N_coll_ than the faunal remains. The offset for carbon is just larger than anticipated trophic level effect, and nitrogen is towards the higher range of values reported for diet–collagen spacing (Bocherens and Drucker 2003; O’Connell et al. 2012). This suggests that the human population may have consumed enriched sources of ^15^N, such as marine or freshwater protein.

### Tooth dentine results for individual QCS1123

**Fig. 4:**
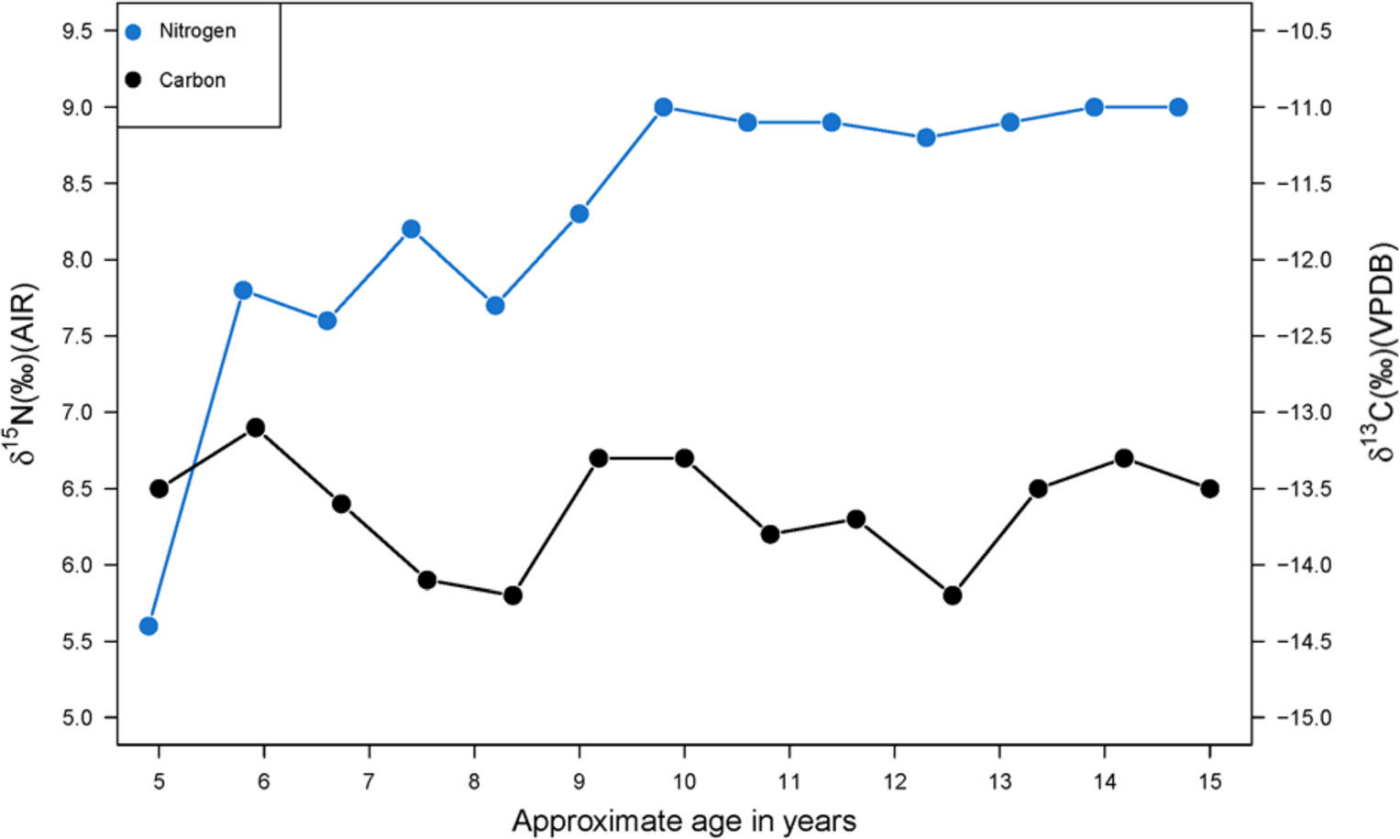
δ^13^C and δ^15^N values for dentine serial sections from second molar (M2) of QCS1123 plotted against approximate age in years. The approximate age assigned to each dentine segment was calculated following Beaumont and Montgomery (2015)

The lower second molar of human individual QCS1123 was serial sectioned to further explore the bulk bone dietary signal (δ^13^C_coll_ of − 13.0‰) that suggested the consumption of C_4_ crops in adulthood. The isotopic profile (Fig. 4) indicates that this individual consumed food similarly enriched in ^13^C throughout their childhood (Table SI1). Analytical uncertainty was ± 0.2‰ (1σ) for both δ^13^C and δ^15^N.

### Bulk hair results for QCS and SB

Bulk hair δ^13^C_ker_ and δ^15^N_ker_ values for QCS are presented in Table 4; incremental hair sample results for SB48 were published in Brown and Alexander (2016) and pooled here for comparison. The individual from SB exhibits a similar δ^15^N signal compared to the QCS individuals but with slight δ^13^C enrichment.

**Table 4:** δ^13^C_ker_ and δ^15^N_ker_ data for bulk hair analysis for adult individuals from QCS and SB

Sample	Sex	Age	%C	δ^13^C_ker_ ‰VPDB	%N	δ^15^N_ker_ ‰AIR	C/N
QCS780	F?	18 + yrs	43.0	− 20.0	14.8	10.8	3.4
QCS845	M	45 + yrs	41.8	− 20.5	14.1	12.1	3.5
QCS2009	F?	18 + yrs	40.7	− 20.1	13.6	12.0	3.5
QCS2018	F	31–45 yrs	44.0	− 20.1	14.8	11.3	3.5
SB48	F	72 yrs	44.7	− 19.1	15.4	11.2	3.4

### Dental calculus

**Fig. 5:**
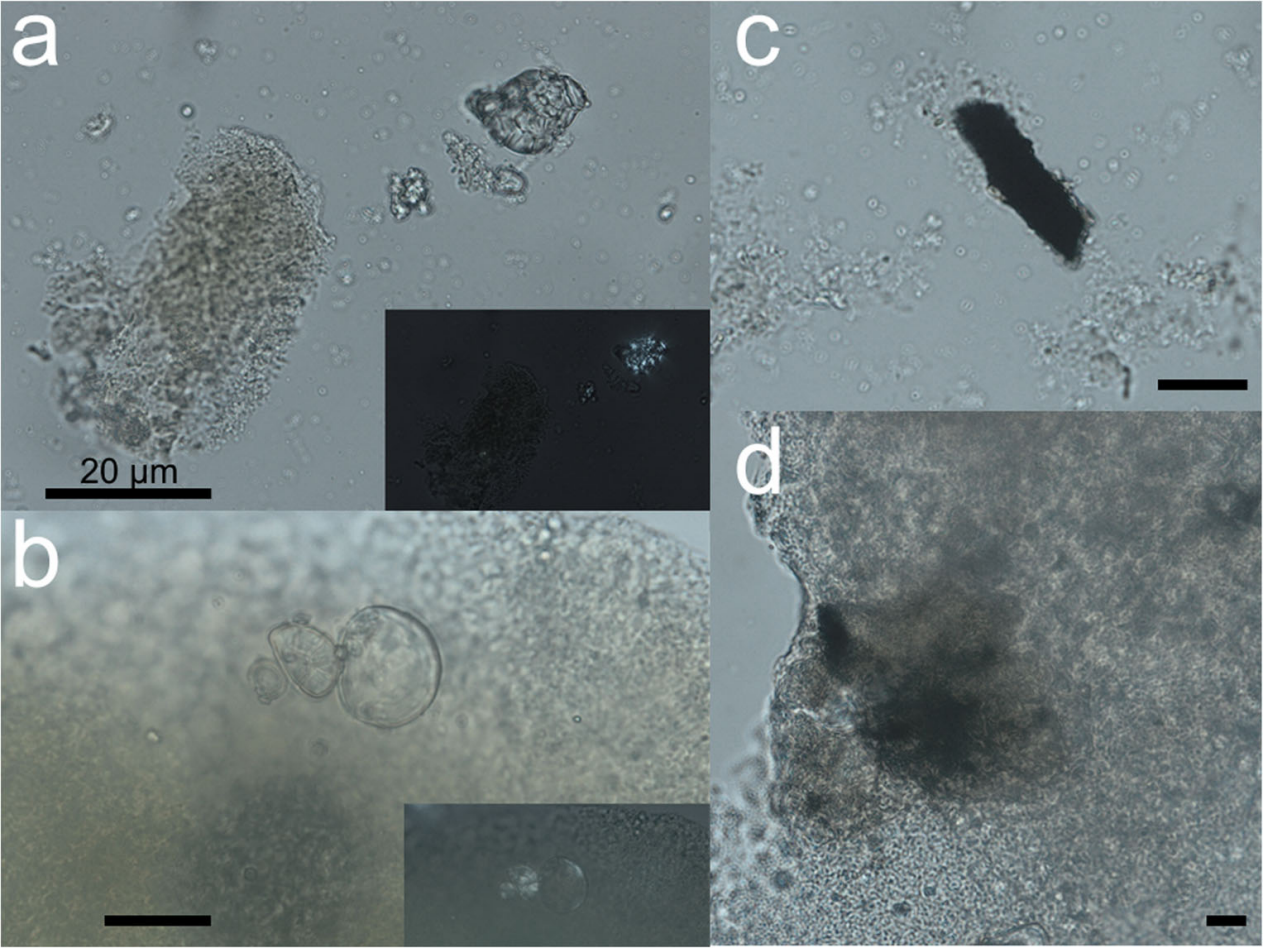
Examples of microremains in dental calculus. (**a**) Starch granules from the tribe Poeae (QCS819), (**b**) starch granules from the tribe Triticeae (QCS427), (**c**) and (**d**) burnt remains (QCS1746, QCS1961, respectively). Note that all microremains are surrounded in dissolving dental calculus matrix

All four QCS individuals were found to have small particles entombed in their calculus. These mainly consisted of mineral grit, undiagnostic plant tissues and starch granules (Table SI2). In general, the microparticle content was low, likely due to the small size of the dental calculus samples available for analysis. In most cases, starch granules were found to be very damaged, and secure identification even to tribe level was not possible. However, the starch granules were of two main typologies: single and compound, hence originating from at least two different sources of starchy plants. Single starch granules were consistent with large, oval to sub-oval granules and small round granules pointing to the bimodal distribution found in the majority of the species of the Triticeae tribe, the tribe of grasses (Poaceae) (Fig. 5) to which wheat and barley belong (Cristiani et al. 2018). Compound starch granules are found in a variety of tribes of grasses and other plants, but, in this context, they could belong to the tribe of Poaceae, and in particular with species of the genus *Avena*; however, precise taxonomic identification was not possible. The most likely source of starch granules of compound typology is likely to be oats, as oatmeal would be a common food at the time. Small fragments of burnt debris, potentially microcharcoal or soot, were also present (Fig. 5). The fragments of plant tissues were too small to allow for taxonomic identification. Overall, the remains were found to be in a very poor status of preservation considering their age and when compared to those of earlier periods (e.g. Cristiani et al. 2018; Hardy et al. 2016; Radini et al. 2017).

## Discussion

### Animal management in post-medieval London

The QCS and PS faunal isotope results are the first reported for post-medieval London and conform to expectations that livestock at this time were raised primarily on C_3_ forage and fodder crops. In contrast, the nitrogen isotope values display an enrichment and variation greater than those seen at other late-medieval and post-medieval British sites (Müldner and Richards 2005, 2007a, 2007b; Millard et al. 2015) and are more typically of the variation seen in modern livestock at a national and regional scale (Camin et al. 2007; Perini et al. 2009; Kelly 2010). The range in δ^15^N is greater than that seen in sixteenth–seventeenth-century Durham (Millard et al. 2015) and likely reflects the great range over which animals were brought to London for slaughter. The improvements in roads and inland waterways, along with the technological developments of steamships and railways, significantly increased the ease and distance with which livestock and “dead meat” were moved, all connecting the surging capital’s population with farmers the length and breadth of the country (Thornbury 1878b; Perren 2000; Turner et al. 2001).

The wide range of δ^15^N_coll_ values observed across this study reflects not only the geographical range from which London’s food supplies were drawn but also the diverse farming practices under which they were raised. At this time of intense agricultural development, there was great diversity in farming practices, with variations in stocking densities, folding and manuring with animal dung and organic waste all having the potential to influence the enrichment and variation seen in these samples (Bogaard et al. 2007; Bateman and Kelly 2007). Some domestic fowl and pigs may also present backyard animals consuming greater quantities of nitrogen-enriched foods (e.g. meat scraps), a phenomenon also reported for medieval pigs from urban York (Müldner and Richards 2007a; Hammond and O’Connor 2013).

### Long-term and short-term dietary signals

The results of this study examine both long- and short-term dietary signals. Comparisons to bone collagen results for London fauna suggest that individuals from both QCS and SB populations ate a long-term diet comprised largely of terrestrial C_3_ foods, with the potential addition of some freshwater or marine resources or C_4_ foods (Ambrose 1993; Schwarcz and Schoeninger 2011). The hair results from the sites of the study provide a snapshot of the final months prior to death and also indicate a largely C_3_ terrestrial-based diet with some input of animal protein. Unfortunately, it was not possible to sample bone from these individuals, which prevents any consideration of a change of diet during the last period of life.

The analysis of dental calculus from four individuals from QCS confirms the consumption of at least two different sources of starchy food, consistent with historical records pointing to the consumption of wheat, rye, barley and oats (Mitchell 1996; Thirsk 2007). Overall, the individuals did not produce a large quantity of identifiable microfossils which, in part, could be due to the poor preservation of the microremains themselves. Retrieved microparticles were very small in size and did not allow for the precise identification of their origin. These findings stand in contrast with previous studies of medieval and post-medieval calculus, which demonstrated higher quantities of identifiable dietary and environmental microdebris (Lazzati et al. 2016; Radini et al. 2016, 2019). Nevertheless, the number of individuals analysed in this study was low. It is possible that the relatively poor preservation of microremains in the QCS calculus is also related to the heavy processing of food as a result of new technological advancements (Clayton and Rowbotham 2009). Analysis of additional post-medieval individuals would be needed to explore this further. Microcharcoal could be the result to the exposure of smoke and has been found consistently in dental calculus samples from a range of periods, including Lower Palaeolithic hominins (Hardy et al. 2016). For this study, it was not possible to analyse calculus from SB for microremains; however, Hendy et al. (2018) identified peptides specific to brassica plants in the calculus of one individual (SB21) providing direct evidence of C_3_ plant consumption.

The range of δ^15^N bulk collagen values within the QCS population may reflect differences in food preference or access. As previously noted, some variation may be due to the large supply network providing London with livestock and meat but a more extensive analysis of post-medieval fauna would be needed to investigate if animals, and ultimately human, isotopic values may reflect geographical differences relating to the supply chain. Dietary variation is also likely to reflect differences in socioeconomic status as QCS is a heterogeneous burial population made up of civilians, patients, prisoners and military personnel (Somerville 1960). Records from the contemporary hospital of St Bartholomew, London, show that patients ate a diet comprised of wheat bread, meat, cheese and ale (Moore 1918; Drummond and Wilbraham 1969). Prisoners would have had a highly restricted diet of bread and water (Howard 1789), and it was not until the late nineteenth century that dietary regulations included foods, such as potatoes and meat (Drummond and Wilbraham 1969). Excluding the outlier QCS1123, six collagen samples from QCS produced δ^15^N values < 11‰, with accompanying δ^13^C values between − 19.2 and − 21.1‰. These δ^15^N values are similar to those seen in some individuals from Kilkenny Workhouse in Ireland who are believed to represent the diet of the rural Irish poor consisting mainly of potatoes (C_3_), with little or no meat protein (Beaumont et al. 2013a). The Scottish poor would have also had a limited diet of oats, pulses and dairy (Collins 1975; Riggs 1994). It is therefore possible that some individuals at QCS may have eaten a diet very low in animal protein or spent most of their life outside of London. A further consideration is that the QCS study population included starving prisoners which could result in higher δ^15^N values that are not representative of long-term diet (Hobson et al. 1993; Fuller et al. 2005; Mekota et al. 2006). Beaumont and Montgomery (2016) used incremental dentine collagen δ^13^C and δ^15^N analyses to identify periods of physiological stress in individuals from Kilkenny workhouse. However, there are no well-documented, associated periods of famine with QCS, and it is therefore harder to confidently attribute changes in δ^15^N to starvation, particularly since the bone collagen of ribs is believed to turnover every 2–5 years (Cox and Sealy 1997).

Pathological evidence of dietary excess among the QCS study group shows that the burial population encompasses a whole spectrum of London society. Two male individuals aged 45 + from QCS were identified as suffering from DISH (Diffuse Idiopathic Skeletal Hyperostosis), a condition that can be associated with obesity (Waldron 1985; Verlaan et al. 2007) and causes the calcification of ligaments and the fusion of the spine (Rogers and Waldron 2001; Roberts and Manchester 2005). The two individuals with DISH produced enriched nitrogen values compared to the mean of the overall population. Individual QCS123 had δ^15^N_coll_ value of 13.2‰, and QCS731 produced a value of 13.8‰. While some studies have shown that some DISH sufferers have elevated δ^15^N_coll_ values, stable isotopes analyses cannot be used to distinguish between those with DISH and unaffected individuals (Müldner and Richards 2007b; Spencer 2008; Quintelier et al. 2014), and it remains difficult to disentangle the influence of physiological and dietary processes. It is, however, possible that these QCS individuals were from the higher ranks of society, and their ^15^N enrichment reflects a high protein diet.

### Military connections

As previously noted, the inclusion of sailors and soldiers in the QCS burial population could potentially be isotopically distinctive from the civilian population due to the relatively high protein intake (Roberts et al. 2012; MacDonald 2014). While it is not possible to match QCS burials directly with the burial records, the presence of specific pathologies or trauma could tentatively identify military personnel. For example, adult male individual QCS117a had a fatal gunshot wound to the head most likely caused by musket fire (Sibun and Ponce 2018). Although suicide cannot be completely ruled out, there are only three documented deaths by gunfire for QCS and only one likely to have involved a musket. The individual in question, Samuel Jackson, was killed for desertion in 1752 (ibid: 87). The isotope results for QCS117a display a slightly enriched δ^15^N_coll_ value of 12.8‰ when compared to the population mean (12.2‰). However, the difference is small and as QCS117 is represented by a cranial fragment the dietary signal probably represents an earlier period of the individual’s life when compared to the majority of the sample population from whom ribs were sampled (Fahy et al. 2017). Another individual (QCS589) had evidence of a severe dislocation of the shoulder resulting in significant bone loss of the humeral head as well as two healed rib fractures. This individual produced a relatively high δ^15^N_coll_ value of 13.6‰, but as their injuries could have been sustained in an accident or confrontation, we cannot confidently identify them as a soldier or sailor.

The heterogeneous social structure of the QCS burial population makes it challenging to confidently identify military personnel based on δ^15^N_coll_ values alone. However, using the δ^13^C_coll_ values, it was possible to identify a dietary outlier. Individual QCS1123 produced a bulk bone carbon value of − 13.1‰ falling within the range of − 16 to −7‰ suggestive of a C_4_-based diet. The individual was a male aged 31–45 years old (Sibun and Ponce 2018), and, given that there was a military presence in the cemetery, his unusual dietary signature may be due to him having originated from elsewhere. To further investigate the dietary signal of QCS1123, dentine from the M2 of the same individual was analysed. The results indicate that they also consumed C_4_ crops throughout childhood and therefore likely migrated to London in later life. QCS1123 was therefore compared to individuals who would be expected to have eaten a substantial amount of C_4_ staples and were buried in North America (Table 5): plantation burials from Virginia in Chesapeake Bay (c.1658–1680) (Ubelaker and Owsley 2003), American soldiers buried at Snake Hill Ontario (1814) (Raynor and Kennett 2008) and British colonial and American soldiers who died at the Battle of Stoney Creek in Ontario (1813) (Emery et al. 2015).

**Table 5:** Mean and standard deviations for δ^13^C_coll_ and δ^15^N_coll_ from adult individuals (18 + years) from Chesapeake, Snake Hill and Stoney Creek (16 + years) (Ubelaker and Owsley 2003; Raynor and Kenneth 2008; Emery et al. 2015)

		δ^13^C_coll_ ‰VPDB				δ^15^N_coll_‰ AIR			
Site	*n*	Mean	SD	Min	Max	Mean	SD	Min	Max
Chesapeake Bay	19	− 16.2	3.2	− 20.5	− 10.5	11.7	1.4	8.7	14.4
Snake Hill	13	− 16.4	1.9	− 17.7	− 15.0	10.4	0.9	8.7	11.9
Stoney Creek	18	− 18.2	2.2	− 20.8	− 13.5	11.1	0.7	9.7	12.3

Individual QCS1123’s carbon value was greater than the mean δ^13^C values for all comparison sites, and only four individuals from Chesapeake had more positive δ^13^C values. Individual QCS1123 is therefore most likely to have spent the majority of their life outside of London. While it is impossible to state definitively if they were associated with the Royal Navy, it is possible that they were recruited or taken prisoner during the American Wars of Independence (1775–1783) and other international conflicts in Northern America (Rink 1986; Rogers 2007; Roberts et al. 2012) or were previously associated with the Royal Naval outposts in the West Indies (Varney 2011). C_4_ cultigens (millet) would also, however, have been available from elsewhere in Europe at this time (Holder et al. 2017).

**Fig. 6:**
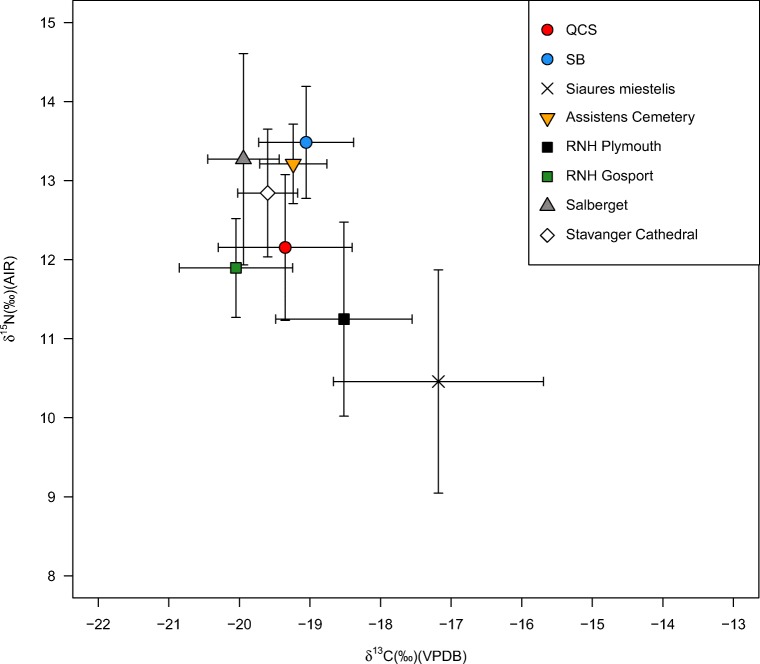
Adult δ^13^C_coll_ and δ^15^N_coll_ mean values and standard deviations from post-medieval Northern European sites of Assistens Cemetery, Denmark (*n* = 111) (Jørkov and Gröcke 2016); Salberget, Sweden (*n* = 32) (Bäckström et al. 2017); Stavanger Cathedral, Norway (*n* = 7) (van der Sluis et al. 2016) and military individuals from Siaures miestelis, Lithuania (*n* = 77) (Holder et al. 2017); Plymouth Hospital, UK (*n* = 38) (Roberts et al. 2012) and the Royal Naval Hospital Gosport, UK (*n* = 24) (Roberts et al. 2012). QCS is marked in red and SB in blue. Adults are defined as individuals 18 years of age or above

To further investigate whether the function of the QCS cemetery as a burial ground for sailors had any isotopic significance, the mean δ^13^C_coll_ and δ^15^N_coll_ values were compared to two contemporary Naval Hospital sites at Plymouth and Gosport (Fig. 6; Table SI3) (Roberts et al. 2012). Individuals from Plymouth Naval hospital exhibited δ^13^C_coll_ mean value towards the expected range for C_4_ crops and included one individual with a substantial input of C_4_ protein. These results correspond with historical evidence that Plymouth population had a higher probability of sailing to North America and therefore a higher likelihood of consuming C_4_ plants or the meat of animals fed on C_4_ foods (Roberts et al. 2012). The QCS population produced mean isotopic values most similar to Gosport. The Gosport population conforms to expectations based on historical literature that seamen ate a controlled diet of C_3_-based foods and a relatively large proportion of meat protein (MacDonald 2004). Despite the inclusion of individual QCS1123 with substantial C_4_ input and the use of the military infirmary of the Savoy during a range of conflicts, such as the American Wars of Independence (1775–1783) and Anglo-American War (1812) which would have provided the opportunity to consume C_4_ foods, the overall majority of the population appears to have eaten a C_3_-based diet.

### Wider comparisons to post-medieval sites

**Fig. 7:**
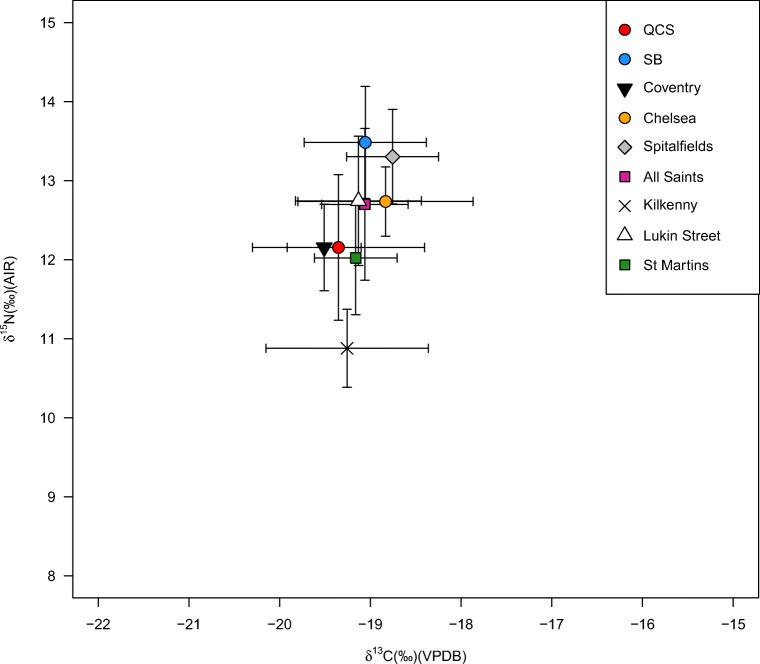
Adult δ^13^C_coll_ and δ^15^N_coll_ mean values and standard deviations for the post-medieval sites of Chelsea (*n* = 28), Lukin Street, London (*n* = 64), Christ Church Spitalfields, London (*n* = 88), All Saints, York (*n* = 10), Coventry (*n* = 11), St Martin’s, Birmingham (*n* = 18) and Kilkenny Workhouse, Ireland (*n* = 14) (Trickett 2006; Beaumont et al. 2013a; Nitsch et al. 2010; Müldner and Richards 2007a; Richards 2006). QCS is marked in red and SB in blue. Adults are defined as individuals being 18 years of age or above

To contextualise the results of this study, the isotopic mean values of QCS and SB were compared to adult bulk bone collagen from other post-medieval populations from across the United Kingdom (Fig. 7; Table SI4) and Northern Europe (Fig. 6; Table SI3). Statistical comparisons were performed between the two sites of this study and published δ^13^C_coll_ and δ^15^N_coll_ results for adult individuals from the London sites of Chelsea, Christ Church Spitalfields and Lukin Street (Table 6) (Molleson et al. 1993; Trickett 2006; Nitsch et al. 2010, 2011). Pair-wise comparisons (Mann–Whitney *U* test) between QCS and Chelsea (*U* = 454.5), Christ Church Spitalfields (*U* = 976) and Lukin Street (*U* = 1498.5) show statistically significant differences in δ^13^C_coll_ (*P* = < 0.03). For δ^15^N_coll_, significant differences were also identified (*P* = < 0.001) between QCS and Chelsea (*U* = 516), Christ Church Spitalfields (*U* = 774) and Lukin Street (*U* = 1303.5).

**Table 6:** Mean and standard deviations for δ^13^C_coll_ and δ^15^N_coll_ from adult individuals (> 18 years) from post-medieval London sites of Chelsea Christ Church Spitalfields and Lukin Street (Trickett 2006; Nitsch et al. 2010; Beaumont et al. 2013a)

		δ^13^C_coll_ ‰VPDB				δ^15^N_ker_ ‰AIR			
Site	*N*	Mean	SD	Min	Max	Mean	SD	Min	Max
Chelsea	28	− 18.8	1.0	− 19.7	− 16.4	12.7	0.4	11.8	13.6
Christ Church Spitalfields	88	− 18.8	0.5	− 19.6	− 16.9	13.3	0.6	12.1	15.3
Lukin Street	64	− 19.1	0.7	− 20.3	− 15.9	12.7	0.8	10.5	14.4

All four sites display an enriched nitrogen mean value when compared to QCS. For Chelsea and Christ Church Spitalfields, this is expected due to the inclusion of wealthier individuals consuming more freshwater or marine food sources and terrestrial animal protein (Molleson et al. 1993; Trickett 2006; Nitsch et al. 2010, 2011). Lukin Street comprises mainly of first- and second-generation Irish immigrants of which some may have been survivors of the Great Irish Famine (Beaumont et al. 2013a). In comparison to other sites from across the United Kingdom, the QCS population fell somewhere between the high and lower class groups. Mean δ^13^C_coll_ and δ^15^N_coll_ values were most similar to those calculated for Coventry (δ^13^C_coll_ − 19.5‰, δ^15^N_coll_ 12.1‰) whom are believed to represent the working classes (Trickett 2006). When the mean bulk δ^15^N_ker_ value for QCS (11.6‰) is compared to hair results from adults from Lukin Street (*n* = 5) (Beaumont et al. 2013a) and Christ Church Spitalfields (*n* = 17) (O’Connell and Hedges 1999), it is the same as the mean value for Lukin Street (11.6‰) and only slightly higher than Spitalfields (11.1‰). The mean δ^13^C_ker_ for QCS is − 20.2‰ compared to mean value of − 19.1‰ for Lukin Street and − 19.4‰ for Christ Church Spitalfields.

When comparing the bulk bone collagen results of SB, there are statistically significant differences (Mann–Whitney *U* test) in δ^15^N_coll_ when compared to Chelsea (*P* = < 0.001, *U* = 122) and Lukin Street (*P* = < 0.001, *U* = 388.5). There was no statistically significant difference in δ^15^N_coll_ when compared to Christ Church Spitalfields (Molleson et al. 1993; Nitsch et al. 2010, 2011). When compared to Northern European and military populations, SB displayed an enriched δ^15^N_coll_ mean value compared to all other assemblages. The likelihood that SB included higher status individuals consuming marine protein is supported by comparison to the δ^15^N_coll_ mean values of Salberget mining community, Sweden, and Assistens cemetery, Copenhagen, which are believed to include individuals consuming appreciable amounts of marine protein (Bäckström et al. 2017) and brackish fish (Jørkov and Gröcke 2016), respectively. The predominantly C_3_-based diet of both the SB and QCS populations is underscored by the clear contrast with the mean δ^13^C_coll_ value for the individuals from Siaures miestelis, Lithuania (Holder et al. 2017). The burial population is believed to include soldiers of Napoleon’s army, including potential recruits from Italy and Poland who would have consumed more C_4_ food sources (Reitsema and Vercellotti 2012; Reitsema et al. 2017).

## Conclusion

The isotopic dietary signatures of human individuals buried at the Queen’s Chapel of the Savoy indicate that the population was very diverse, complementing historical and osteological datasets which suggest parishioners, seamen, soldiers, patients and prisoners were buried at the site. The differences exhibited in δ^15^N_coll_ values likely reflect the differences in diet due to social class (access to different amounts of meat and freshwater/marine protein) and occupation (military and civilian). Furthermore, the cemetery’s association with a prison and hospital means some individuals’ δ^15^N_coll_ values may have been influenced by physiological processes. The analysis of faunal remains from the same site and a comparable London site enabled the human isotopic data to be contextualised and suggest that the human population is more likely to have consumed terrestrial protein than marine sources. The results of this study provide further insights into animal management and dietary variation among London populations during the post-medieval period. The faunal results are the first reported for post-medieval London and demonstrate that animals were primarily raised on C_3_ fodder. The nitrogen values show greater variability suggesting different feeding regimes of different species, as well as livestock being drawn from other parts of the United Kingdom and being bought to London for market (Woodward 1973, 1977; Galloway 2012). While the human dental calculus samples provided limited dietary information, the poor preservation of plant remains could be evidence of change in food technology and should be further investigated using a larger dataset.

The comparative data from St Barnabas reveals further differences in social class and that this population is likely to have included more wealthy individuals consuming larger quantities of animal protein. Comparisons were also made between the Queen’s Chapel of the Savoy with other post-medieval sites which revealed an overlap of mean δ^15^N_coll_ values in comparison to London populations and military and Northern European populations, underscoring the diversity of the burial population. We have shown that the general overall range of δ^13^C_coll_ values fits well with expectations of the largely C_3_-based terrestrial diet typical for post-medieval Britain. However, the bulk bone collagen and dentine data for QCS1123 demonstrate that the population likely also included migrants, who originated from regions where C_4_ plants were more readily available for human consumption and as graze for animals. This study demonstrates how isotopes can be used to further our understanding of differences in social status and mobility within burial populations during the post-medieval period.

## Electronic supplementary material

ESM 1 (XLSX 17 kb)
